# Stronger linkage of diversity-carbon decomposition for rare rather than abundant bacteria in woodland soils

**DOI:** 10.3389/fmicb.2023.1115300

**Published:** 2023-03-02

**Authors:** Hui Cao, Suying Li, Huan He, Yaoqin Sun, Yichao Wu, Qiaoyun Huang, Peng Cai, Chun-Hui Gao

**Affiliations:** State Key Laboratory of Agricultural Microbiology, State Environmental Protection Key Laboratory of Soil Health and Green Remediation, College of Resources and Environment, Huazhong Agricultural University, Wuhan, China

**Keywords:** rare versus abundant bacteria, stochasticity versus determinism, carbon decomposition, environmental adaptability, environmental breadth, phylogenetic signal

## Abstract

Soil microbial diversity is important for maintaining ecosystem functions. However, the linkage between microbial diversity, especially rare and abundant bacterial diversity, and carbon decomposition remains largely unknown. In this study, we assessed the establishment and maintenance of rare and abundant bacterial α-diversities at the taxonomic and phylogenetic levels and their linkages with soil carbon decomposition separately in four Chinese woodlands. Compared to abundant bacteria, rare bacteria showed higher community diversity, tighter phylogenetic clustering, wider environmental breadth, stronger phylogenetic signals, and higher functional redundancy. The assembly of the abundant bacterial subcommunity was governed by stochastic (59.2%) and deterministic (41.8%) processes, whereas the assembly of the rare bacterial subcommunity was mainly dominated by deterministic processes (85.8%). Furthermore, total phosphorus, soil pH, and ammonium nitrogen balanced stochastic and deterministic processes in both rare and abundant bacterial subcommunities. Our results reveal that rare bacteria displayed stronger environmental adaptability and environmental constraint. Importantly, the α-diversities of rare taxa, rather than abundant taxa, were significantly related to carbon decomposition. This study provides a holistic understanding of biogeographic patterns of abundant and rare bacteria and their α-diversities in relation to carbon decomposition, thus helping us better predict and regulate carbon dynamics under the background of global climate change.

## 1. Introduction

Woodlands, one of the stable terrestrial carbon sinks, play a significant role in the global carbon cycle and consequently affect global climate change (Dixon et al., 1994; Bonan, 2008). Dynamic changes in woodland carbon pools are induced by carbon assimilation and decomposition (Dan et al., 2020). The carbon decomposition of woodland releases enormous amounts of carbon dioxide (CO_2_), increasing global warming (Rustad et al., 2000). Soil microorganisms (e.g., arbuscular mycorrhizal fungi and *Acidobacteria*) are the main engines of carbon decomposition and their microbial diversity is vital for maintaining ecosystem functions (Trivedi et al., 2016; Liang et al., 2017; Zhang et al., 2018; Tláskal and Baldrian, 2021). Therefore, understanding the relationship between microbial diversity and carbon decomposition would be helpful in the mitigation of global climate change.

Microorganisms usually show skewed abundance distributions, consisting of some species with higher abundance (abundant taxa) and most species with lower abundance (rare taxa; Nemergut et al., 2011). The abundant taxa are traditionally considered to dominate microbial communities and perform major ecological functions (Pedrós-Alió, 2012). Recent studies have shown a significant positive correlation between rare species diversity and multifunctionality, which may be attributed to a limited microbiota performing a specific ecological function (Chen et al., 2019; Zhang et al., 2022). For instance, *Desulfosporosinus* spp. is the most critical sulfate-reducing agent in peatlands, but its relative abundance is only 0.0006% (Pester et al., 2010). In acid mine drainage, rare bacteria (e.g., *Acidithiobacillus* sp. FKB1 and FKB2) are closely related to the processes of nitrogen fixation and sulfur oxidation (Hua et al., 2015). However, the relative contribution of rare and abundant bacterial diversity to the functions possessed by such a broad range of microorganisms as carbon decomposition is unclear.

Microbial diversity maintenance would be effectively evaluated by the microbial environmental adaptability and ecological assembly process. Environmental adaptability, which was clarified by the environmental breadth and phylogenetic signal, reflects the resistance of species to environmental change (Goberna and Verdú, 2016). The abundant and rare taxa show different abilities of environmental adaptability. For instance, abundant rather than rare microbial taxa show stronger environmental adaptability in wetland soils of the Qinghai-Tibet Plateau (Wan et al., 2021a). Ecological assembly processes, including stochastic and deterministic processes, shape the composition and coexistence pattern of microbial community and couple microbial community function with ecosystem function (Feng et al., 2018). Both stochastic and deterministic processes influence bacterial community assemblies (Gao et al., 2020; Jiao and Lu, 2020b). Specifically, stochastic processes determine the assembly of abundant bacterial subcommunity, whereas deterministic processes dominate the assembly of rare ones in Chinese farmland soils (Jiao and Lu, 2020b). Yet, few works have revealed the ecological assembly process and environmental adaptability of rare and abundant bacteria in woodland ecosystems.

We chose four woodlands across eastern China and collected soils covering four soil types, showing distinct differences in physicochemical properties (Supplementary Table S1). This study set out to i) evaluate the linkage of rare and abundant taxa with carbon decomposition in woodlands and ii) reveal the environmental adaptability and assembly process of rare and abundant bacterial subcommunities. Since microbial diversity is closely connected to ecosystem function (Tilman et al., 2014; Morris et al., 2020), the contributions of rare and abundant bacterial diversity to carbon decomposition should both exist. Given the highly competitive and metabolic capacity of abundant bacteria (Jousset et al., 2017), we hypothesized that abundant bacteria would have greater environmental fitness than those of rare bacteria and may contribute more to carbon decomposition in soils. To test our hypotheses, we performed 16S rRNA gene sequencing and determined the enzymatic activities related to carbon decomposition.

## 2. Materials and methods

### 2.1. Soil collection

We chose four woodlands covering four soil types (i.e., black, brown, cinnamonic, and red soils), and these four woodlands were mainly covered with poplar, poplar, poplar and pine trees, respectively. The four sites were separately located in Qiqihar in Heilongjiang Province (124°78′E, 48°19′N), Laiyang in Shandong Province (120°43′E, 36°58′N), Fengqiu in Henan Province (114°33′E, 35°2′N), and Qiyang in Hunan Province (111°53′E, 26°46′N; Supplementary Figure S1). Four sampling sites (50 × 50 m) were established in each woodland. Soil cores were taken from the soil surface (0–20 cm) at each sampling site using the five-point sampling method. The samples were immediately covered with ice packs and sent back to the laboratory within 24 h. Impurities such as plant and animal residues, stones and roots were manually removed. Then, the samples were passed through a sieve with a 2 mm pore size. A portion of the samples was stored for physicochemical properties and enzyme activities determination and the remainder were kept at -80°C for DNA extraction.

### 2.2. Soil physicochemical properties and enzyme activities determination

Standard analytical methods were followed to measure thirteen soil physicochemical indicators (Bao, 2000), including soil moisture content (Moi), water holding capacity (WHC), pH, soil organic matter (SOM), total carbon (TC), total nitrogen (TN), nitrate-nitrogen (NO_3_^−^-N), ammonium nitrogen (NH_4_^+^-N), total phosphorus (TP), total potassium (TK), available phosphorus (AP) and available potassium (AK) and electrical conductivity (EC). Details of the assays were given in the supplementary information (Supplementary Method).

Enzyme activities related to carbon cycling, including β-1,4-glucosidase (βG), β-D-cellobiohydrolase (CB), β-xylosidase (XYL) and α-glucosidase (αG), were measured using 4-methylumbelliferone (MUB)-linked substrate by a microplate reader (Spark; Tecan, Switzerland; Marx et al., 2001; Bell et al., 2013). The carbon cycling index (CCI) was determined by employing Z-score transformation (Luo et al., 2018; Wan et al., 2021c) based on the following equation. The equation is EV_i_ = (E_i_ – E_ave_)/SD_i_, where EV is the standardized variable for factor i (i.e., TC, βG, CB, XYL, and αG), E_i_ is the factor i, and E_ave_ and SD_i_ are the mean value and standard deviation of factor i over all samples, respectively. The CCI is the mean of the EV values for the five selected factors.

### 2.3. DNA extraction and 16S rRNA gene sequencing

The DNeasy^®^ PowerSoil^®^ Kit (Qiagen, Germantown, MD) was used to extract total microbial genome DNA from 0.25 g soil. The extracted DNA was quantified using a NanoDrop NC2000 spectrophotometer (Thermo Fisher Scientific, Waltham, MA, United States).

The forward primer 338F (5′-ACT CCT ACG GGA GGC AGC A-3′) and the reverse primer 806R (5′-GGA CTA CHV GGG TWT CTA AT-3′) were employed to amplify the V3-V4 region of bacterial 16S rRNA genes (Mori et al., 2014; Wan et al., 2022). The following PCR procedure was used: 98°C for 5 min, followed by 25 cycles of denaturation at 98°C for 30 s, annealing at 53°C for 30 s, and extension at 72°C for 45 s, with a final extension of 5 min at 72°C. PersonalBio (Shanghai, China) performed Illumina MiSeq sequencing. The raw sequences were imported into QIIME2 and the “DADA2” plugin was used for denoising and quality control, resulting in amplicon sequence variants (ASVs; Bolyen et al., 2019; Prodan et al., 2020). The Greengene 13_8 99% database (DeSantis et al., 2006; McDonald et al., 2012) was used as a reference to classify and annotate the ASVs using the QIIME 2 feature classifier plugin (Bokulich et al., 2018). The sequencing datasets were deposited in the SRA database^1^ under the accession numbers SRR22803208–SRR22803223.

### 2.4. Data analysis

First, we deleted ASVs (excluding archaea and fungal ASVs) with few than 20 reads (Jiao and Lu, 2020a). Then, ASVs with a relative abundance >0.1% of total sequences were assigned to “abundant taxa,” and ASVs with a relative abundance <0.01% of total sequences were assigned to “rare taxa,” according to previous definition criteria (Wan et al., 2021b,c).

To evaluate the phylogenetic clustering, the standardized effect sizes of the mean nearest taxa distance (SES.MNTD) were determined based on the null model using the “ses.mntd” function of the “picante” R package (Kembel et al., 2010). The β-mean nearest taxon distance (βMNTD) was obtained *via* the “comdistnt” function of the “picante” package to quantify the abundance-weighted phylogenetic distance between the two closest ASVs in the two communities (Kembel et al., 2010). Ordinary least squares between phylogenetic similarity and geographic distance was used to characterize the distance attenuation relationship. Spearman’s correlation was implemented with the “vegan” package (Dixon, 2003) in R software (version 4.2.2).

Environmental breadths were assessed by the response thresholds of rare and abundant bacteria to the environmental gradient, which was implemented using the “TITAN2” package (Baker and King, 2010). The ecological preferences of species were obtained by calculating their correlations with environmental variables. Subsequently, we determined the phylogenetic signals by the Fritz-Purvis D test (Goberna and Verdú, 2016). Based on 16S rRNA gene sequence similarity, functional predictions were carried out using “Tax4Fun2” package, and functional redundancy indices (FRI) were calculated for different functions (Wemheuer et al., 2020).

The β-nearest taxon index (βNTI) and Bray–Curtis-based Raup-Crick (RCbray) were used to evaluate rare and abundant bacterial subcommunity assemblies based on the null model (Chase and Myers, 2011). When |βNTI| > 2, the selection effect dominates the difference in community composition. Among them, if βNTI < −2, it is considered that homogeneous selection leads to a more similar community phylogeny, and if βNTI > +2, it is considered variable selection. When |βNTI| < 2, the community composition differences are due to dispersal limitation, homogenizing dispersal, or undominant processes. If RCbray < −0.95, it indicates that homogenizing dispersal plays a decisive role in community assembly, and if RCbray >0.95, it is mainly dispersal limitation; −0.95 < RCbray <0.95 is used to estimate the undominant assembly process, including weak selection role and dispersal, diversification and drift process (Stegen et al., 2013). The correlation between physicochemical factors and βNTI was determined by the Mantel test.

## 3. Results

### 3.1. General distribution pattern of rare and abundant bacteria

**Figure 1:**
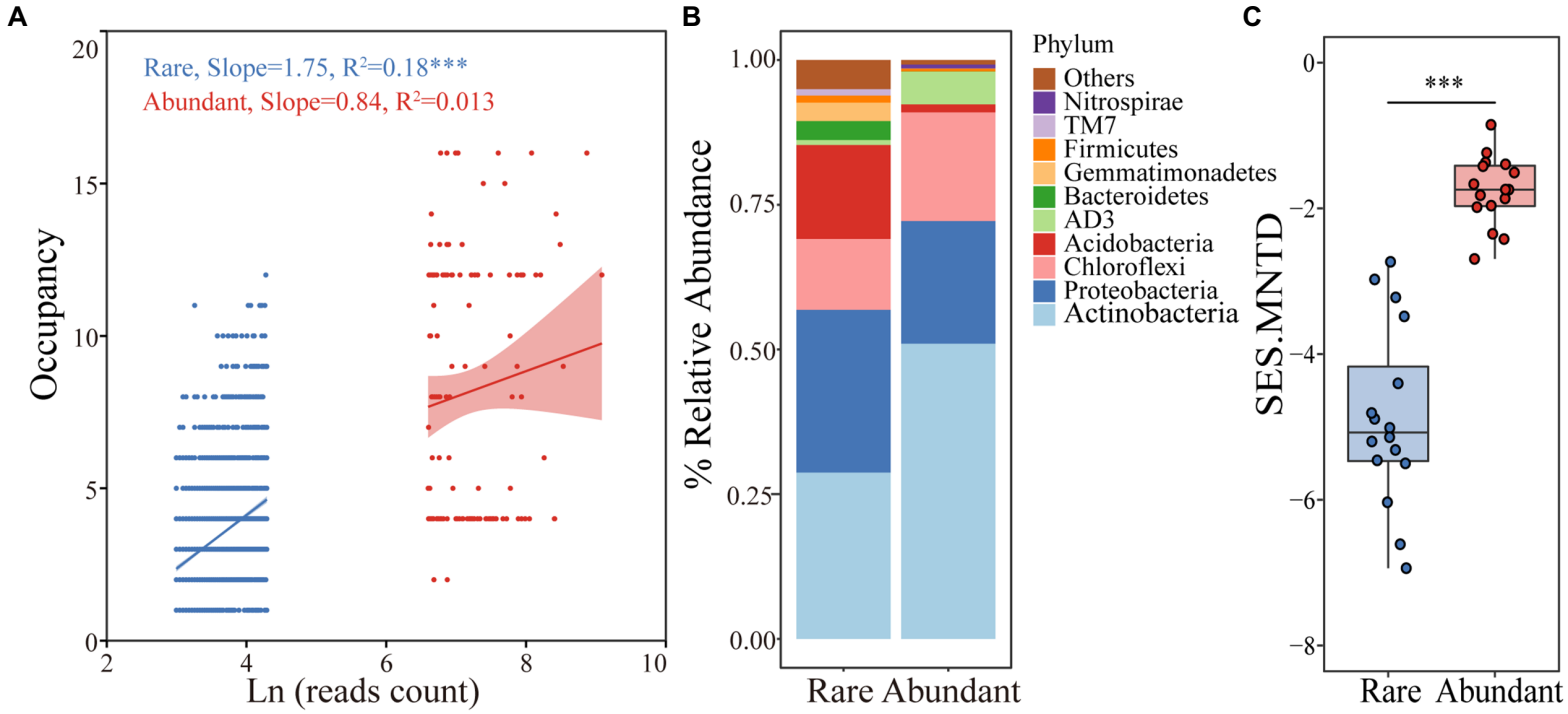
Distribution patterns of rare and abundant taxa in woodland soils. **(A)** Relationships between abundance and occupancy of rare and abundant ASVs. **(B)** Distributions of rare and abundant bacteria at the phylum level. **(C)** Comparison of the differences in SES.MNTD values between rare and abundant bacteria.

A total of 831,328 reads were divided into 6,360 ASVs (ASVs with <20 reads were deleted). Rare (3,356 ASVs) and abundant taxa (132 ASVs) contributed 52.8% and 2.1% of the total community richness, with total relative abundances of 1.57% and 25.1%, respectively. Occupancy was significantly and positively related to abundance for rare bacteria (R^2^ = 0.18, *p* < 0.001) but not for abundant bacteria (R^2^ = 0.013, *p* > 0.05; Figure 1A). In more than half of all the samples, 52.3% of the abundant taxa and 3% of the rare taxa were detected. Abundant taxa were assigned to 8 phyla and rare taxa to 26 phyla. The abundant taxa mainly included *Actinobacteria* (51%), *Proteobacteria* (21.1%) and *Chloroflexi* (18.8%), while *Actinobacteria* (28.7%), *Proteobacteria* (28.1%), *Acidobacteria* (16.3%) and *Chloroflexi* (12.3%) were dominant in the rare taxa (Figure 1B). These findings revealed significant differences in the rare and abundant bacterial distribution patterns. Rare taxa had noticeably lower mean values of SES.MNTD than those of abundant taxa (*p* < 0.001, Wilcoxon rank-sum test), with all values less than 0 (Figure 1C), indicating that rare taxa have a tighter phylogenetic distribution than that of abundant one. According to the Mantel test for correlations between environmental variables and bacterial subcommunities, soil moisture, pH and TP were significantly correlated with community diversity and composition (Table 1).

**Table 1:** Pearson’s correlations between environmental factors and **α**-diversities, PERMANOVA showing the pure effect of each environmental factor on community composition, and Mantel’s test of environmental variables against the **β**NTI of rare and abundant bacterial subcommunities.

Factor	Taxonomic α-diversity		Phylogenetic α-diversity		Community composition		Community assembly	
	Rare	Abundant	Rare	Abundant	Rare	Abundant	Rare	Abundant
Longitude	0.147	0.591*	0.154	0.281	27.13%***	41.50%***	0.315*	0.486**
Latitude	0.231	0.355	0.235	0.189	26.445***	45.15%***	0.425**	0.586**
Moi	−0.891***	0.591*	−0.887***	0.885***	24.99%***	38.85%***	0.360**	0.137
WHC	−0.839***	0.728***	−0.833***	0.940***	23.11%**	33.43%***	0.272*	0.006
pH	0.960***	−0.611*	0.955***	−0.775***	27.77%***	51.73%***	0.909**	0.670**
EC	−0.001	0.463	0.002	0.390	25.09%***	37.14%***	0.085	0.255*
TC	−0.081	0.186	−0.078	0.352	22.76%**	25.86%*	−0.114	0.002
SOM	−0.523*	0.552*	−0.523*	0.739***	22.79%**	26.65%**	−0.102	−0.039
TN	−0.488	0.792***	−0.479	0.771***	24.63%**	28.73%**	−0.122	−0.144
NH_4_^+^-N	−0.901***	0.290	−0.901***	0.597*	26.88%***	55.26%***	0.898**	0.861**
NO_3_^-^-N	−0.146	0.584*	−0.136	0.524*	24.39%**	33.28%***	−0.118	0.052
TP	0.955**	−0.499*	0.956***	−0.724**	27.97%***	55.53%***	0.961**	0.799**
AP	0.340	0.466	0.349	−0.077	25.19%***	25.33%*	0.082	0.147
TK	−0.399	0.665**	−0.391	0.706**	23.70%**	28.76%*	−0.179	−0.098
AK	0.023	0.375	0.027	0.345	24.70%***	36.25%**	0.084	0.241*

Abbreviations are defined in the “Materials and Methods” section. Asterisks denote significance (*, *p* < 0.05; **, *p* < 0.01; ***, *p* < 0.001).

**Figure 2:**
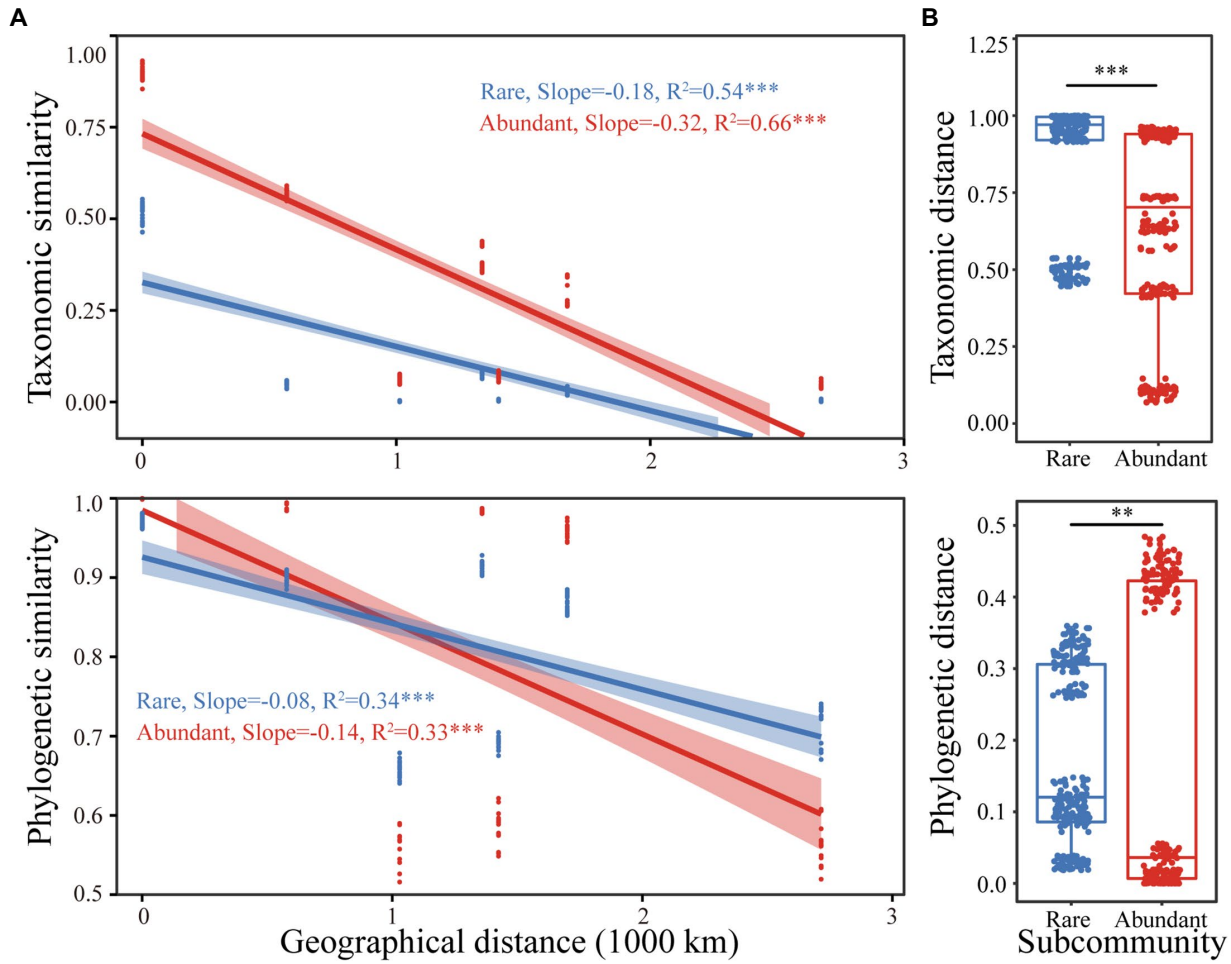
**(A)** The relationships between geographical distance and the taxonomic and phylogenetic similarity of rare and abundant bacteria. **(B)** Boxplots show differences in taxonomic and phylogenetic distances between rare and abundant taxa. Asterisks indicate significance (**, *p* < 0.01, ***, *p* < 0.001).

Both rare and abundant taxa exhibited distance decay relationships, as their taxonomic similarities were significantly negatively correlated with geographic distances. However, the attenuation coefficients of the two were different. The abundant taxa’s attenuation coefficient (0.32) was larger than the rare taxa’s (0.18), suggesting that abundant taxa had a higher species turnover rate. Similarly, the phylogenetic similarities of the rare and abundant bacteria significantly decreased with increasing geographical distances. The abundant taxa (0.14) had a larger attenuation coefficient than that of rare taxa (0.08; Figure 2A). The taxonomic and phylogenetic distances of rare bacterial subcommunities were remarkably higher than those of abundant ones (*p* < 0.01 for both; Figure 2B). Furthermore, significant correlations were observed between taxonomic and phylogenetic distances of both abundant and rare bacterial subcommunities. The correlation was stronger for the abundant bacterial subcommunity (Supplementary Figure S2), implying that abundant and rare bacterial subcommunity phylogenies were differentially sensitive to environmental changes.

### 3.2. Linkages between carbon cycling index and rare and abundant bacterial diversities

**Figure 3:**
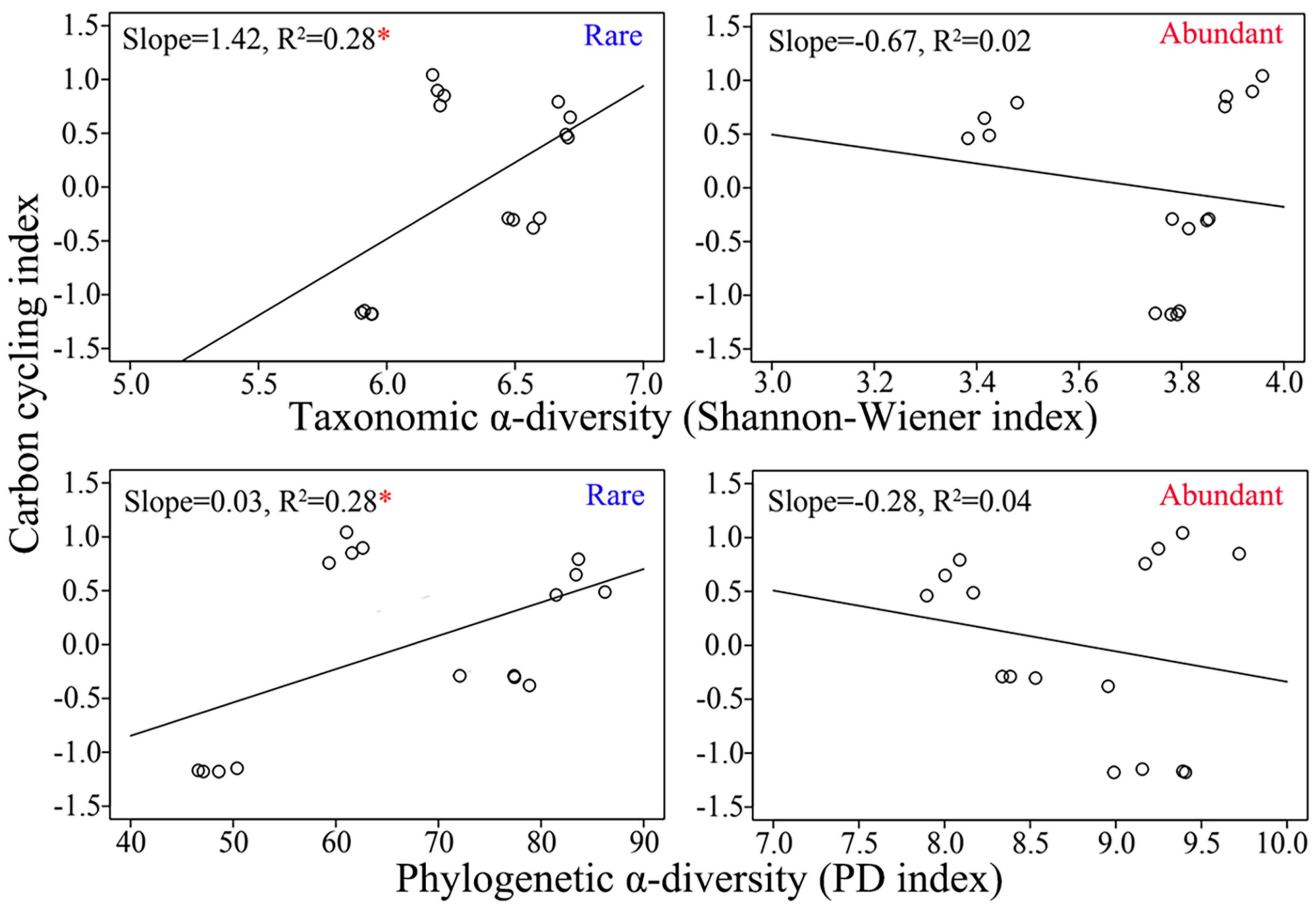
Correlations between the carbon cycle index and α-diversity of rare and abundant taxa. The asterisk indicates significance (*, *p* < 0.05).

The relative abundances of the top 20 ASVs of abundant taxa were more significantly correlated with the activities of carbon-cycling-related enzymes, whereas the relative abundances of the top 20 ASVs of rare taxa were more significantly correlated with TC and SOM (Supplementary Figure S3). Both the Shannon–Wiener index and PD index were higher for rare bacteria than for abundant bacteria (Supplementary Figure S4). The taxonomic α-diversity of rare taxa was significantly positively related to the carbon cycle index (R^2^ = 0.28, *p* < 0.05), while the taxonomic α-diversity of abundant taxa was insignificantly negatively related to the carbon cycle index (R^2^ = 0.02, *p* > 0.05; Figure 3). Similarly, there was a significantly positive correlation between rare taxa’s phylogenetic α-diversity and carbon cycle index (R^2^ = 0.28, *p* < 0.05), whereas there was an insignificant negative correlation between abundant taxa’s phylogenetic α-diversity and carbon cycle index (R^2^ = 0.04, *p* > 0.05; Figure 3). The above results indicated that the α-diversities of rare and abundant taxa were related differently to the carbon decomposition function.

### 3.3. Environmental adaptability and subcommunity assembly processes of rare and abundant taxa

**Figure 4:**
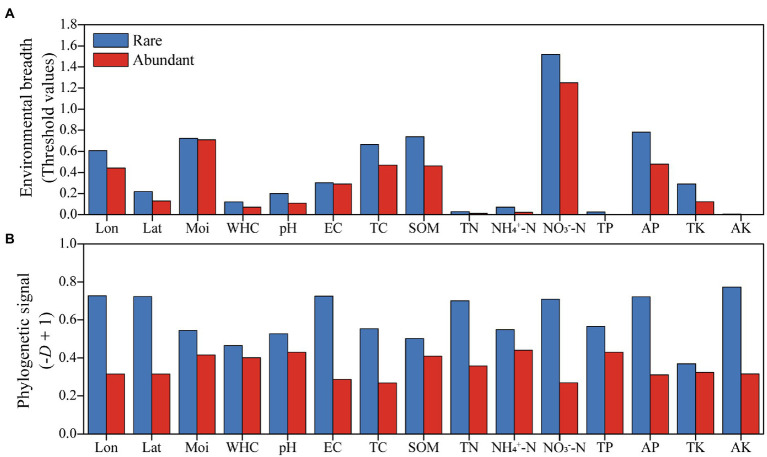
Environmental adaptability of abundant and rare bacteria in woodland soils. **(A)** Environmental breadths were assessed by the response thresholds of rare and abundant bacteria to environmental factors. **(B)** Phylogenetic signals reflect the conservation of environmental preference traits in rare and abundant taxa. Abbreviations for environmental variables were described in the “materials and methods” section.

Environmental threshold analysis was employed to investigate the responses of rare and abundant taxa to each geospatial and physicochemical variable tested. Rare taxa showed broader environmental thresholds for all factors tested compared to abundant taxa (Figure 4A). According to the Fritz-Purvis D test, rare taxa had stronger phylogenetic signals than those of abundant taxa for all environmental variables examined (Figure 4B).

**Figure 5:**
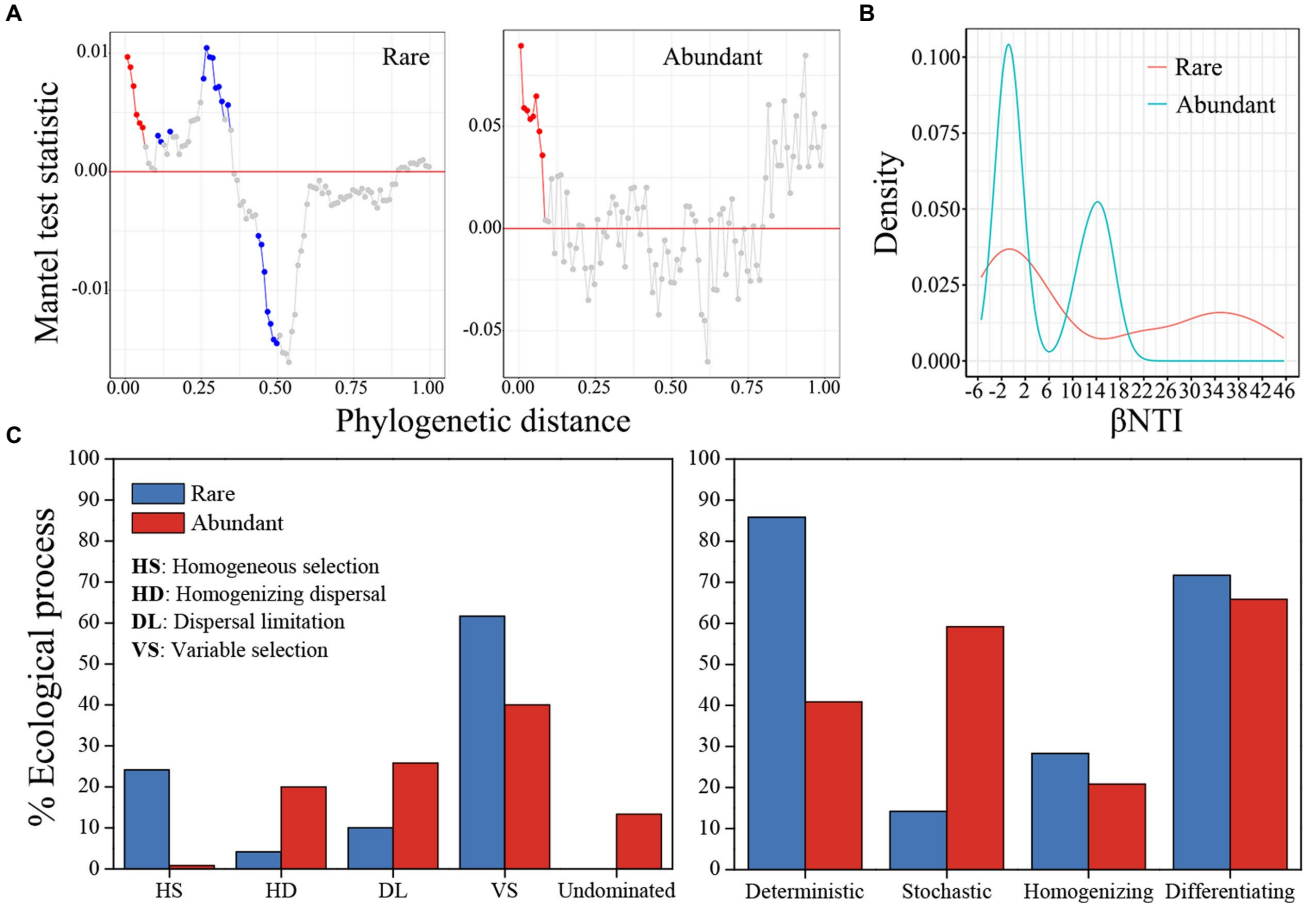
The rare and abundant bacterial subcommunity assembly. **(A)** Phylogenetic Mantel correlogram evaluating phylogenetic signals in rare and abundant bacterial subcommunities. Each point denotes the Mantel correlation coefficient between phylogenetic distances and ASV niche differences. Colors indicate significance (red, *p* < 0.01, blue, *p* < 0.05 and gray, *p* > 0.05). **(B)** Kernal density estimates for βNTI distributions for rare and abundant bacteria. **(C)** Null model-based ecological assembly processes of rare and abundant bacterial subcommunities.

In both rare and abundant bacterial subcommunities, the phylogenetic Mantel correlogram displayed a significant correlation between ASV niche differences and phylogenetic distance along with phylogenetic distance (*p* < 0.05; Figure 5A). The median βNTI values were 4.371 for rare taxa and-0.198 for abundant taxa (Figure 5B). Null model analysis results showed that ecological processes contributed differently to rare and abundant bacterial subcommunity assemblies (Figure 5C). Variable selection (61.7%) had the largest contribution to the subcommunity assembly of rare bacteria, but variable selection (40%) and dispersal limitation (25.8%) contributed most to the subcommunity assembly of abundant bacteria. Homogeneous selection, homogenizing dispersal and undominated processes had little influences on rare and abundant bacterial subcommunity assemblies. Therefore, deterministic (85.8%) and differentiating (71.7%) processes governed the subcommunity assembly of rare bacteria, while the processes of differentiating (65.8%) and stochastic (59.2%) dominated abundant bacterial subcommunity assembly. The value of the sorting to dispersal limitation for rare taxa was approximately 8.58, which was greater than the value of 1.58 for abundant taxa, indicating that rare taxa were subject to stricter environmental constraints than abundant ones. According to Mantel tests, pH, NH_4_^+^-N and TP were more closely linked with βNTI than other physicochemical variables (Table 1).

### 3.4. The function of rare and abundant bacterial subcommunities

**Figure 6:**
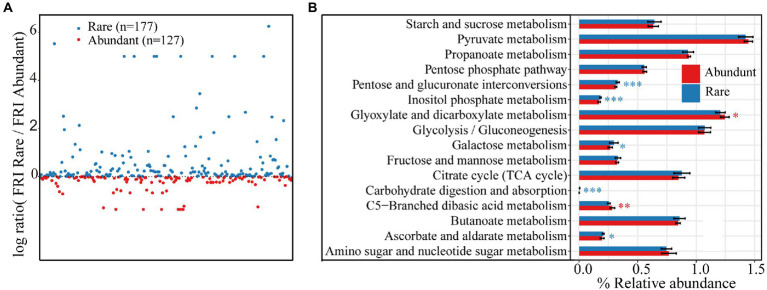
**(A)** Functional redundancy of rare and abundant taxa at KEGG pathway level 3. **(B)** Differences in functional redundancy among 16 carbon decomposition functions between rare and abundant bacteria. Asterisks indicate significance (*p < 0.05; **p < 0.01; ***p < 0.001).

At KEGG pathway level 3, there were a total of 304 functions based on the function prediction results. The rare taxa showed higher functional redundancy (177 functions) than the abundant taxa (127 functions; Figure 6A). Some carbon catabolic functions were significantly higher for rare rather than abundant taxa, including pentose and glucuronate interconversions, inositol phosphate metabolism, galactose metabolism, carbohydrate digestion and absorption, and ascorbate and aldarate metabolism. However, glyoxylate and dicarboxylate metabolism and C5-Branched dibasic acid metabolism were higher in abundant bacteria than in rare bacteria (Figure 6B). More significant correlations were found between carbon-cycling-related enzyme activity and carbon-cycling-related functions of abundant bacterial subcommunities than rare ones (Supplementary Figure S5). However, significant correlations were observed almost only between total carbon, soil organic matter and carbon-cycling-related functions of rare bacterial subcommunities.

## 4. Discussion

### 4.1. Stronger linkage between rare bacterial diversity and carbon decomposition

The rare and abundant bacterial subcommunities had various species compositions in the woodland ecosystem. Rare bacterial subcommunity had much higher taxonomic and phylogenetic α-diversity than those of abundant bacterial subcommunity, supporting the view that rare bacteria are pivotal to α-diversity (Lynch and Neufeld, 2015). “Environmental filtration” affects bacterial diversity and thus the ecological functions of the bacterial community (O’Malley, 2008). Here, soil moisture, pH and TP are the most critical factors influencing the rare and abundant bacterial subcommunity diversity and structure in woodland soils. pH is closely correlated with microbial community composition in woodland soils (Borchers et al., 2022). Additionally, an earlier study reported that TN and pH affected the abundant bacterial subcommunity composition, whereas organic carbon shapes the rare bacterial subcommunity composition in oil-contaminated soils (Jiao et al., 2017). The discrepancies may be due to differences in geographical and environmental factors.

Soil carbon decomposition depends on microbial activity and diversity (Monteux et al., 2020; Xu et al., 2021). Carbon-cycling-related functions of abundant and rare taxa showed different potential effects on carbon-decomposing-related enzymes and soil organic matter. This phenomenon might be due to the large population of abundant bacteria and the high redundancies of some carbon-cycling-related functions of rare bacteria. Microbial diversity can respond to changes in environmental factors and serve as a predictor of ecosystem multifunctionality. Recent research has shown that rare taxa are the main driving factors for ecosystem multifunctionality (Chen et al., 2019; Zhang et al., 2022). Furthermore, rare bacteria are proven to be more central to the nitrogen and phosphorus cycles than abundant bacteria (Wei et al., 2019; Liang et al., 2020). Our research showed a stronger linkage of diversity-carbon decomposition for rare rather than abundant bacteria in woodland soils. The different linkages of rare and abundant bacterial diversity with carbon decomposition might be attributed to distinct mechanisms for their establishment and maintenance. Future studies will validate these findings on large spatial and long temporal scales for better generalization.

### 4.2. Stronger environmental adaptability of rare bacteria

Recently, researchers have focused on the environmental adaptability of rare and abundant taxa in farmland, grassland, wetland and other ecosystems (Ji et al., 2020; Jiao and Lu, 2020a; Wan et al., 2021a,c). Nevertheless, there are few studies on the environmental adaptability of rare and abundant bacteria in woodland soils. Contrary to our hypothesis, rare bacteria had a wider environmental breadth than that of abundant bacteria for all physical and chemical factors, indicating that rare taxa can effectively use a broader range of resources in woodlands. This could be explained by the fact that rare bacteria have a higher richness than that of abundant bacteria, allowing a large number of specialists to adapt to environments and thus obtain a broader environmental breadth (Pedrós-Alió, 2012). This finding differs from the case in which abundant species adapt to a wider environmental gradient than rare species in eastern China farmland (Jiao and Lu, 2020a). The discrepancy may be caused by different kinds of microorganisms and relative abundances of rare subcommunities. Similar to several previous studies, abundant bacteria were more omnipresent in woodland soils than rare bacteria and could be detected in almost all samples (Ji et al., 2020; Jiao and Lu, 2020b). Most rare bacteria were found in only a few soil samples, consistent with previous results (Jousset et al., 2017), which may be due to their low competitiveness and metabolic capacity, limiting their habitat breadth (Barberán et al., 2014).

Rare bacteria exhibited stronger phylogenetic signals than those of abundant bacteria, consistent with a finding in coastal wetlands (Gao et al., 2020), indicating that closely related species have more similar ecological preferences in rare taxa. The functional characteristics of microorganisms based on ecological preferences are greatly influenced by their evolutionary history, in contrast to the limited influence of environmental heterogeneity (Thomas et al., 2016; Saladin et al., 2019). For instance, the growth and metabolism of soil microorganisms are affected more by evolutionary history than by environmental variables such as temperature and precipitation in four forest habitats (Morrissey et al., 2019). Interestingly, a stronger positive correlation was observed between taxonomic distance and phylogenetic distance for abundant bacteria than for rare bacteria (Supplementary Figure S2). This may be owing to the fact that the phylogeny of rare microbial subcommunities is less susceptible to persistent environmental changes (Ji et al., 2020). The potential potentiality of a community to maintain phylogeny can reflect its ability to conserve ecological niches (Miller et al., 2013). Hence the decoupling between taxonomic and phylogenetic distances suggests that rare bacteria maintain ecological niches better than abundant bacteria. These findings probably explain why rare bacteria had a wider environmental breadth and a unique biogeographic pattern. In short, the above two findings indicated that rare bacteria were more environmentally adaptable than abundant bacteria in woodland soils at the level of taxonomy and phylogeny, which may be a crucial reason for the stronger linkage of diversity-carbon decomposition for rare rather than abundant bacteria.

### 4.3. Distinct ecological assembly processes shaping rare and abundant bacterial subcommunities

Deterministic processes (mainly variable selection) dominated rare bacterial subcommunity assembly, while stochastic processes (e.g., dispersal limitation) contributed more to abundant bacterial subcommunity assembly in woodland soils. This is consistent with the finding that abundant taxa are more environmentally constrained in mountain forests in northern Xinjiang, China (Wang et al., 2021). Nevertheless, rare and abundant bacterial subcommunity assemblies in eastern Chinese farmland are dominated by homogeneous selection belonging to deterministic processes (Jiao and Lu, 2020b). Rare bacterial subcommunity assembly in Tibetan Plateau grassland was mainly controlled by stochastic processes, whereas abundant bacterial subcommunity assembly was governed by both stochastic and deterministic processes (Ji et al., 2020). These inconsistent results might be due to geographic heterogeneity and different environmental adaptations of microorganisms (Shi et al., 2018). In addition, the lower SES.MNTD of rare taxa in our study indicates tighter phylogenetic clustering, consistent with prior research (Jiao and Lu, 2020a; Wan et al., 2021c). An earlier study have documented that environmental filtering mediates phylogenetic clustering in microbial communities (Horner-Devine and Bohannan, 2006). This seems to be confirmed by our findings that rare taxa subject to more variable selection controls are more phylogenetically clustered.

Soil pH, NH_4_^+^-N and TP played vital roles in rare and abundant bacterial subcommunity assemblies. In-depth studies more recently have reported that pH balances deterministic and stochastic processes in rare and abundant bacterial subcommunities of Chinese farmland (Jiao and Lu, 2020b) and dryland montane forest soils (Wang et al., 2021). Additionally, pH strongly affected community assemblies of bacteria in grassland soils (Wang et al., 2017) and functional bacteria (e.g., aerobic methanotrophs) in forest soils (Li et al., 2021). The decisive role of pH in shaping bacterial community assembly may be attributable to its strong influence on cell growth and metabolism. A small change in 1.5 units of *in-situ* pH reduces by 50% bacterial activity (Fernández-Calviño and Bååth, 2010). Ammonium nitrogen, another critical factor in bacterial community assembly, was supported by another study (Wan et al., 2021a). Soil NH_4_^+^-N may mediate microbial community assembly by affecting soil microbial activity or altering soil bulk density (Pan et al., 2018). In addition, it is worth noting that TP was momentous. Phosphorus increases total soil carbon by promoting plant growth, thereby increasing microbial biomass and changing community structure. On the other hand, phosphorus can also affect microbial growth by changing the pH and osmotic pressure of the soil (Huang et al., 2016). Considering the coupling process of element cycles such as carbon, nitrogen, and phosphorus (Mooshammer et al., 2017), more elements may be involved in mediating rare and abundant bacterial subcommunity assemblies. In the future, we will consider more environmental samples and physicochemical factors to verify our conjecture at larger spatial scales.

## 5. Conclusion

Our study explored the diversity, environmental adaptations and community assembly mechanisms of rare and abundant bacteria in woodland soils and their correlations with carbon decomposition. Rare taxa had more community diversity, higher phylogenetic signal, wider environmental breadth, tighter phylogenetic cluster and more environmental constraints than those of abundant ones. Rare bacterial diversity was strongly related to carbon decomposition at taxonomic and phylogenetic levels, and its functional redundancy contributed more to carbon cycle-related functions. These findings are helpful in better understanding rare and abundant bacterial diversity maintenance and predicting carbon dynamics under global climate change.

## Data availability statement

The datasets presented in this study can be found in online repositories. The names of the repository/repositories and accession number(s) can be found in the article/Supplementary material.

## Author contributions

HC: conceptualization, methodology, formal analysis, visualization, and writing – original draft. SL: investigation, methodology, and data curation. HH and YS: writing – review and editing. YW: supervision, and writing – review and editing. QH: resources and supervision. PC and C-HG: resources, supervision, funding acquisition, and writing – review and editing. All authors contributed to the article and approved the submitted version.

## Funding

This work was supported by the National Natural Science Foundation of China (42177281 and 32100090), Royal Society-Newton Advanced Fellowship (NAF\R1\191017), and Fundamental Research Funds for the Central Universities (2662021JC012 and 2662022ZHQD001).

## Conflict of interest

The authors declare that the research was conducted in the absence of any commercial or financial relationships that could be construed as a potential conflict of interest.

## Publisher’s note

All claims expressed in this article are solely those of the authors and do not necessarily represent those of their affiliated organizations, or those of the publisher, the editors and the reviewers. Any product that may be evaluated in this article, or claim that may be made by its manufacturer, is not guaranteed or endorsed by the publisher.

## Footnotes

^1^ https://trace.ncbi.nlm.nih.gov
