# Supplementary material for: Stronger linkage of diversity-carbon decomposition for rare rather than abundant bacteria in woodland soils
Source: Front Microbiol. 2023 Mar 2;14:1115300. doi: 10.3389/fmicb.2023.1115300 (PMC10017465; doi:10.3389/fmicb.2023.1115300)
Supplement: Supplementary file 1 [file Data_Sheet_1.pdf]

Supplementary Information

Stronger linkage of diversity-carbon decomposition for rare rather than abundant bacteria in woodland soils

Hui Cao, Suying Li, Huan He, Yaoqin Sun, Yichao Wu, Qiaoyun Huang, Peng Cai, Chun-Hui Gao*

State Key Laboratory of Agricultural Microbiology, State Environmental Protection Key Laboratory of Soil Health and Green Remediation, College of Resources and Environment, Huazhong Agricultural University, Wuhan 430070, China

*To whom correspondence should be addressed: Dr. Chun-Hui Gao, Phone: +86 27 87 671033, E-mail: gaoch@mail.hzau.edu.cn

Running title: Rare taxa mediate carbon decomposition

Supplementary Method: Soil physicochemical properties determination

The soil moisture content (Moi) was determined by the drying method. Soil water holding capacity (WHC) was determined by using the chamber gauze method. Soil pH was determined using a pH meter (PHS-3E; INESA, China) with a soil-to-water ratio of 1:2.5. Soil electrical conductivity (EC) was determined using a conductivity meter (DDS-11A; Leici, China). Soil organic matter (SOM) was determined using the potassium dichromate external heating method. Soil total nitrogen (TN) and total carbon (TC) were determined using an elemental analyzer (Vario PYRO cube and Isoprime100; Elementar, Germany). Soil nitrate nitrogen ($NO3^{-}$-N) and ammonia nitrogen ($NH4^{+}$-N) were extracted with 2 mol/L potassium chloride at a ratio of 1:5 and then determined by a flow injection analyzer (AA3; SEAL, Germany). Total phosphorus (TP) and total potassium (TK) were digested by perchloric acid-concentrated sulfuric acid, followed by the phosphorus molybdenum blue method for TP and the flame spectrophotometer (M410; Sherwood, England) for TK. Soil available phosphorus (AP) was extracted with sodium bicarbonate and then measured by the phosphorus molybdenum blue method. Soil available potassium (AK) was extracted with ammonium acetate and then measured by flame spectrophotometer (M410; Sherwood, England Sherwood M410).

Table S1 Geographical location and physicochemical properties of the tested soil samples.

	Woodland 1	Woodland 2	Woodland 3	Woodland 4
Soil type	black soil	brown soil	cinnamon soil	red soil
Latitude (N)	48°19′	36°58′	35°2′	26°46′
Longitude (E)	124°78′	120°43′	114°33′	111°53′
Moi（%）	24.14±0.507 a	14.50±0.011 c	14.74±0.077 c	23.07±0.130 b
WHC（%）	77.32±0.555 a	50.67±7.111 b	41.62±0.622 b	72.42±8.688 a
pH	6.47±0.00 c	7.11±0.08 b	8.70±0.07 a	4.26±0.03 d
EC（mS/m）	52.4±1.0 a	18.9±0.7 b	17.4±1.1 b	6.7±0.1 c
TC（g/kg）	22.21±0.018 a	10.05±0.011 d	15.11±0.012 b	11.54±0.003 c
SOM（g/kg)	45.67±1.057 a	21.94±1.289 c	24.18±1.650 c	30.63±1.458 b
TN（g/kg）	1.99±0.003 a	1.11±0.004 c	0.76±0.002 d	1.18±0.002 b
NH_4^+-N（mg/kg）	4.67±0.107 b	4.23±0.108 c	4.28±0.113 c	5.62±0.247 a
NO_3^--N（mg/kg）	378.82±21.222 a	105.71±0.887 b	39.80±4.665c	34.02±4.749 c
TP（g/kg)	0.55±0.006 c	0.61±0.003 b	0.70±0.013 a	0.34±0.012 d
AP（mg/kg）	13.40±1.252 b	32.05±1.418 a	3.27±0.473 c	0.00±0.000 d
TK（g/kg)	9.50±0.484 a	3.40±0.265 bc	2.64±0.433 c	3.75±0.409 b
AK（mg/kg）	379.04±3.654 a	126.92±0.000 c	154.32±3.164 b	61.14±3.164 d

Note: Abbreviations are defined in the "Materials and Method" section.

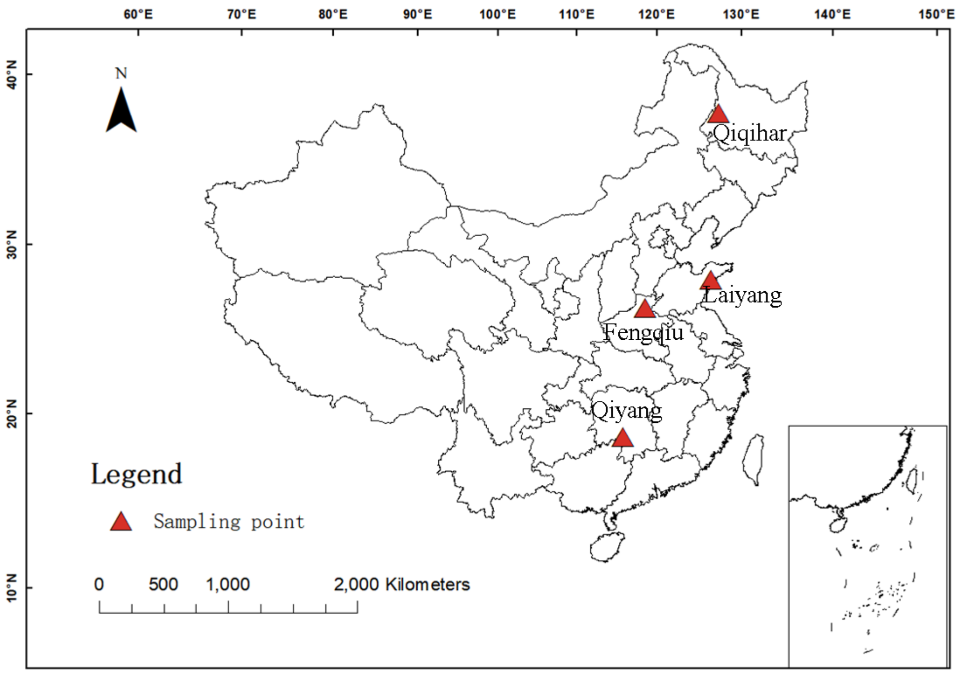

Fig. S1 Distribution of woodland soil collection sites.

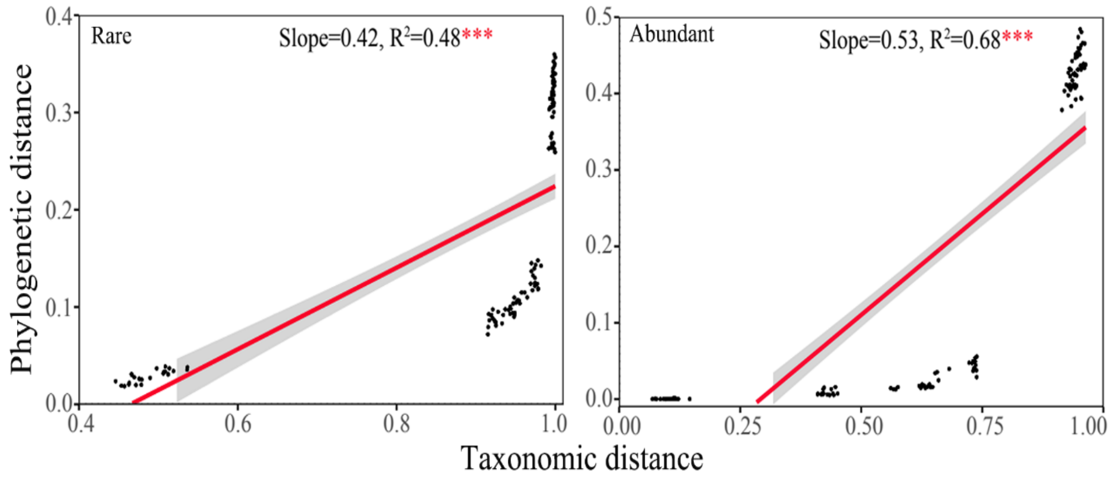

Fig. S2 Correlation of taxonomic distance with phylogenetic distance of rare and abundant taxa. The asterisk indicates significance (***, P <0.001).

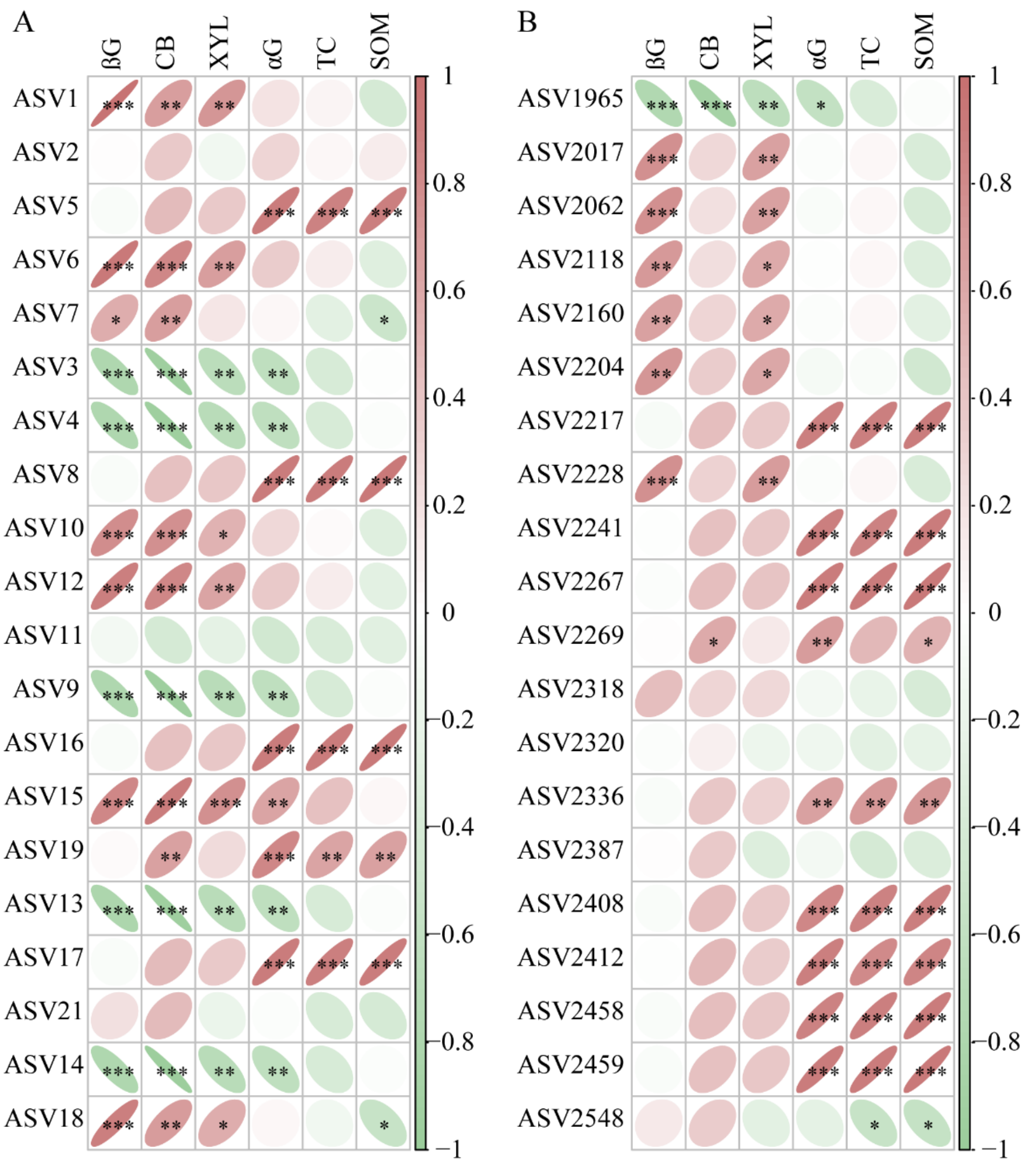

Fig. S3 Correlation heatmap of the relative abundance of ASVs in the top 20 of abundant (A) and rare bacteria (B) and cycling-related enzyme activities, total carbon and soil organic matter. The legend indicated the color range of different r values and the asterisks indicate significance (*P < 0.05; **P < 0.01; ***P < 0.001).

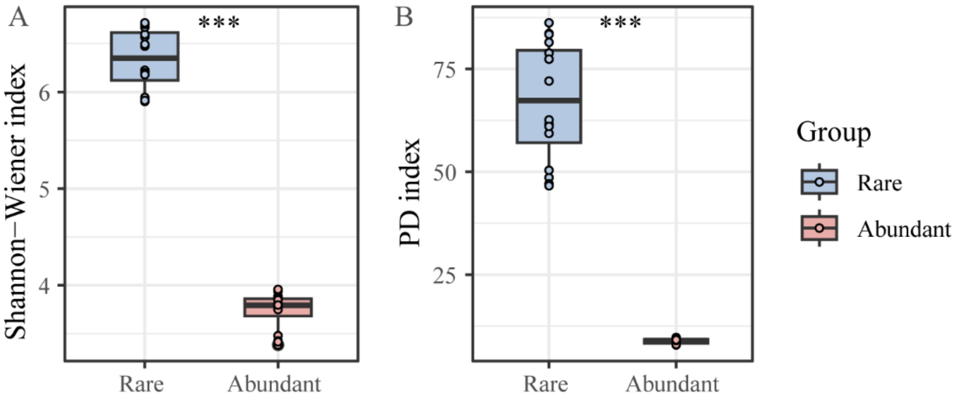

Fig. S4 Comparison of the differences in the Shannon–Wiener index and PD index between rare and abundant bacteria. The asterisk indicates significance (***, *P* <0.001).

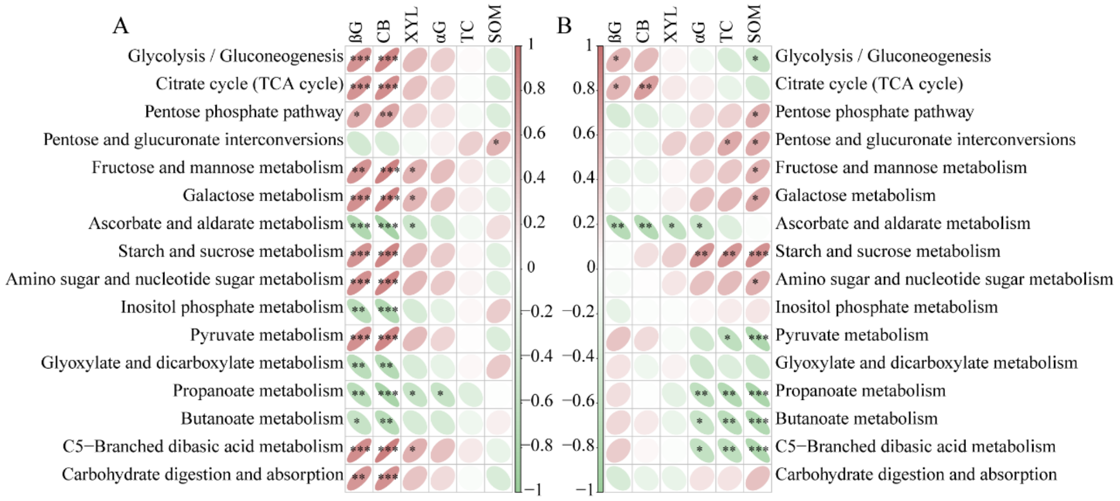

Fig. S5 Correlation heatmap of carbon-cycling-related functions of abundant (A) and rare taxa (B) and carbon-cycling-related enzyme activities, total carbon and soil organic matter. The legend indicated the color range of different r values and the asterisks indicate significance (**P* < 0.05; ***P* < 0.01; ****P* < 0.001).
